# Cholangiocarcinoma: spectrum of appearances on Gd-EOB-DTPA-enhanced MR imaging and the effect of biliary function on signal intensity

**DOI:** 10.1186/s12885-015-1039-x

**Published:** 2015-02-06

**Authors:** Shi-Ting Feng, Ling Wu, Huasong Cai, Tao Chan, Yanji Luo, Zhi Dong, Keguo Zheng, Zi-Ping Li

**Affiliations:** 1 Department of Radiology, The First Affiliated Hospital, Sun Yat-Sen University, 58th, The Second Zhongshan Road, Guangzhou, Guangdong 510080 China; 2 Medical Imaging Department, Union Hospital, Hong Kong, 18 Fu Kin Street, Tai Wai, Shatin, NT Hong Kong

**Keywords:** Cholangiocarcinoma, Gd-EOB-DTPA, Magnetic resonance imaging, Biliary function

## Abstract

**Background:**

To describe the Gd-EOB-DTPA-enhanced MRI appearances of cholangiocarcinoma, and evaluate the relative signal intensities (RSIs) changes of major abdominal organs, and investigate the effect of total bilirubin (TB) levels on the RSI.

**Methods:**

25 patients with pathologically-proven cholangiocarcinoma underwent Gd-EOB-DTPA-enhanced MRI. The visualization of the biliary system during biliary phase (BP) was observed. RSIs of the abdominal aorta (A), portal vein (V), liver (L), and spleen (S) were measured.

**Results:**

On hepatocellular phase (HP), exophytic tumors (*n* =10) and infiltrative tumors (*n* =10) were hypointense, polypoid tumors (*n* = 2) were hypointense, and combined type tumors (*n* = 3) had mixed appearances. While patients with normal TB levels (≤22 μmol/L, *n* = 12) had clear visualization of the biliary tree during BP, those with elevated TB levels (>22 μmol/L, *n* = 13) had obscured or no visualization. In addition, patients with normal TB levels had higher RSI_A_, RSI_V_ and RSI_S_ than those with elevated TB levels on all dynamic phases (*P <*0.001), and lower RSI_A_, RSI_V_ and RSI_S_ on HP and BP (*P <*0.001). Patients with normal TB levels had higher RSI_L_ than those with elevated TB levels on all phases (*P* <0.001).

**Conclusions:**

RSIs of major abdominal organs reflected underlying biliary function. Cholangiocarcinoma patients with elevated TB levels had delayed excretion of Gd-EOB-DTPA compared with patients with normal TB levels.

## Background

Cholangiocarcinoma is a primary malignancy that arises from both intra- and extrahepatic bile duct epithelium with various growth patterns. The typical growth pattern of cholangiocarcinoma can be categorized into four types: exophytic (mass-forming), infiltrative (periductal), polypoid (intraductal), and combined (a combination of the above) [1]. It is the most common primary malignancy of the biliary tree, accounting for 15% of all liver cancers [2,3]. Gross resection of the tumour with concomitant partial hepatectomy is currently the most effective treatment [4,5].

The advantages of MR imaging using gadolinium diethylenetriamine pentaacetic acid (Gd-DTPA) in the detection and diagnosis of cholangiocarcinoma have been well documented [6-8]. Gadolinium ethoxybenzyl diethylenetriamine pentaacetic acid (Gd-EOB-DTPA) is an amphipathic derivative of Gd-DTPA (i.e., Gd-DTPA with a covalently bound lipophilic ethoxybenzyl moiety) [9]. It combines the features of conventional extracellular contrast agents and hepatocyte-specific contrast agents. Previous studies have demonstrated the superiority of Gd-EOB-DTPA in detecting and characterizing lesions in patients with known or suspected focal or diffuse liver disease [10,11]. However, few studies have evaluated its value for detection of cholangiocarcinoma [12-15]. In addition, while biliary excretion of Gd-EOB-DTPA is known to provide positive T1-weighted intrabiliary contrast imaging [16-18], only a few studies have evaluated MR cholangiography with Gd-EOB-DTPA.

Consequently, the primary aim of this study is to describe the Gd-EOB-DTPA-enhanced MR imaging appearances of different types of cholangiocarcinoma that might differ from previously known features on Gd-DTPA -enhanced MR imaging, and to quantitatively evaluate the relative signal intensity (RSI) of major abdominal organs during different phases and investigate the effect of total bilirubin (TB) levels on the RSI.

## Methods

### Patients

The study was conducted in accordance with ethical guidelines for human research and was compliant with the Health Insurance Portability and Accountability Act (HIPAA). As such, the study received IRB or ethical committee approval, and that written informed consent was obtained from all patients.

Fifty-three patients with suspected cholangiocarcinoma underwent Gd-EOB-DTPA-enhanced MR imaging from November 2011 to July 2012 at our institute. Subsequently, 28 of those initially selected were excluded from the study for one of following reasons: unavailable pathological examination (*n* = 10); received treatment in the interval between MR imaging and laboratory examination (*n* = 9).; insufficient image quality due to motion artifacts (*n* = 7); or unusual pathological results (papilloma; *n* = 2).

The remaining 25 patients who had a histological diagnosis of cholangiocarcinoma constituted the study population. Eighteen patients were treated with bile duct resection, hepatectomy, and hepaticojejunostomy; three were treated with bile duct excision only; two underwent orthotopic liver transplantation; and two underwent percutaneous biopsy but no further surgery. The patients included 18 men and seven women with a mean age of 53.7 years (range 23-75 years). Six patients had underlying chronic hepatitis B viral infection and two had liver cirrhosis due to hepatitis B infection. None of the patients had undergone treatment for malignancy at the time of the MR imaging.

### MR Imaging

MR examinations were performed on a 3.0 T MR clinical imager (Magnetom Trio; Siemens Medical Systems, Erlangen, Germany) with the use of an eight-channel phased-array surface coil. Patients underwent a fasting period of four hours before the examination. Prior to contrast medium administration, all patients were imaged with the following unenhanced sequences: T1-weighted, fat-suppressed fast low-angle shot (FLASH; TR/TE 235/2.2 ms, flip angle 70°, slice thickness 6 mm, matrix 240 × 320, FOV 328 × 350); in-phase and out-of-phase T1-weighted gradient echo (GRE; TR/TE 200/3.7 ms and 200/2.2 ms, flip angle 65°, slice thickness 6 mm, matrix 256 × 192, FOV 328 × 350); and T2-weighted, half-Fourier single-shot rapid acquisition with relaxation enhancement (HASTE; TR/TE 1600/91 ms, refocusing angle 150°, slice thickness 5 mm, matrix 320 × 320, FOV 360 × 360). Each patient received the standard dose, 0.025 mmol/kg of body weight, of Gd-EOB-DTPA (Primovist, Bayer Schering Pharma, Berlin, Germany) as an intravenous bolus injection, which was administered at a rate of 2 mL/s. The bolus injection was followed by a 20 mL saline flush.

A post-contrast, T1-weighted, 3D spoiled GRE sequence with chemically-selective fat suppression (TR/TE 3.3/1.2 ms, flip angle 13°, slice thickness 2 mm, matrix 128 × 256, FOV 328 × 350) was performed at 25 seconds after contrast medium injection in the arterial phase (AP), at 66 seconds in the portal venous phase (PVP), at 180 seconds in the equilibrium phase (EP), at 20 minutes in the hepatocellular phase (HP), and at 50 minutes in the biliary phase (BP) [also known as Gd-EOB-DTPA-enhanced MR cholangiography, or EOB-MRC].

### Image analysis

All MR images were retrospectively reviewed by two experienced abdominal radiologists at a workstation (Siemens Leonardo Syngo 2009B). Both radiologists were aware of the diagnosis of cholangiocarcinoma but were blinded towards patient's medical history, laboratory examination, and pathological details.

### Qualitative image analysis

The radiologists reached a consensus regarding the following features: tumor morphology (mass-forming, infiltrative, polypoid or combined), tumor location and extent, signal intensity compared with a normal liver, signal homogeneity, enhancement pattern, and associated findings, including ductal dilatation, hepatic metastases, tumor thrombus, lobar atrophy, and visualization of the biliary system.

### Quantitative image analysis

Mean signal intensities (SIs) of the abdominal aorta (A), portal vein (V), liver (L), spleen (S), and erector spinae (E) were measured by the two radiologists using circular regions of interest (ROI; ranging from 100 to 6000 mm^2^) at the level of porta hepatis on AP, PVP, EP, HP and BP images. The relative signal intensity (RSI) was calculated as follows: RSI_X_ = mean SI_X_/mean SI_E_.

Patients were divided into two groups according to the serum total bilirubin (TB) level: a normal TB (NTB) group (TB level ≤22 μmol/L) and an elevated TB (ETB) group (TB level >22 μmol/L).

### Statistical analysis

Statistical analysis was performed using SPSS 13.0 for Windows (Statistical Package for Social Science, Chicago, USA). Comparisons between the RSI_A_, RSI_V_, RSI_L_, and RSI_S_ in both groups were performed by using the Student’s *t*-test. A *P* value of < .05 was considered to indicate a statistically significant difference.

## Results

### Qualitative analysis

**Figure 1:**
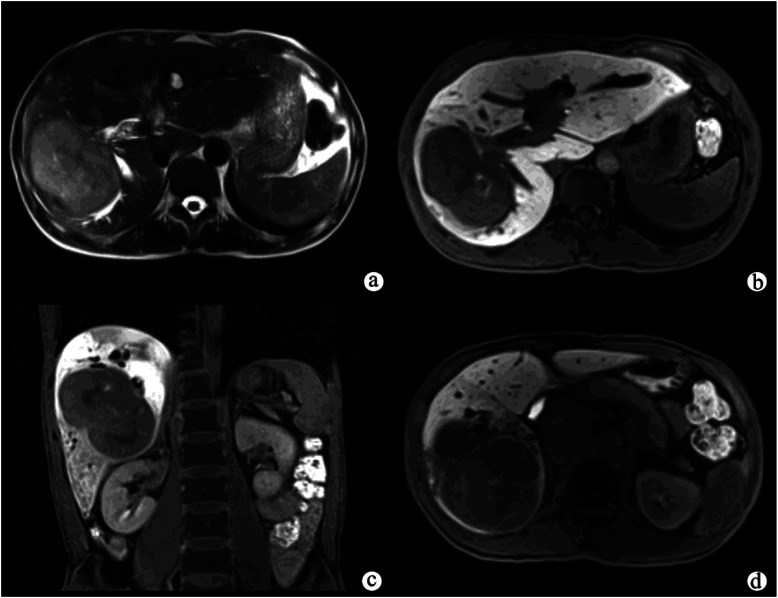
**Exophytic peripheral cholangiocarcinoma in a patient with abdominal pain.** A T2-weighted image **(a)** showed a large, hyperintense mass with lobulated margins in the right hepatic lobe, and the adjacent bile duct was dilated. The hepatocellular phase **(b, c)** showed that the tumor was inhomogeneously hypointense compared with the liver parenchyma. The liver parenchyma also showed inhomogeneous enhancement on the hepatocellular phase **(c)**. The biliary phase **(d)** showed that the common bile duct was completely filled with Gd-EOB-DTPA, indicating that the function of biliary system was normal.

**Figure 2:**
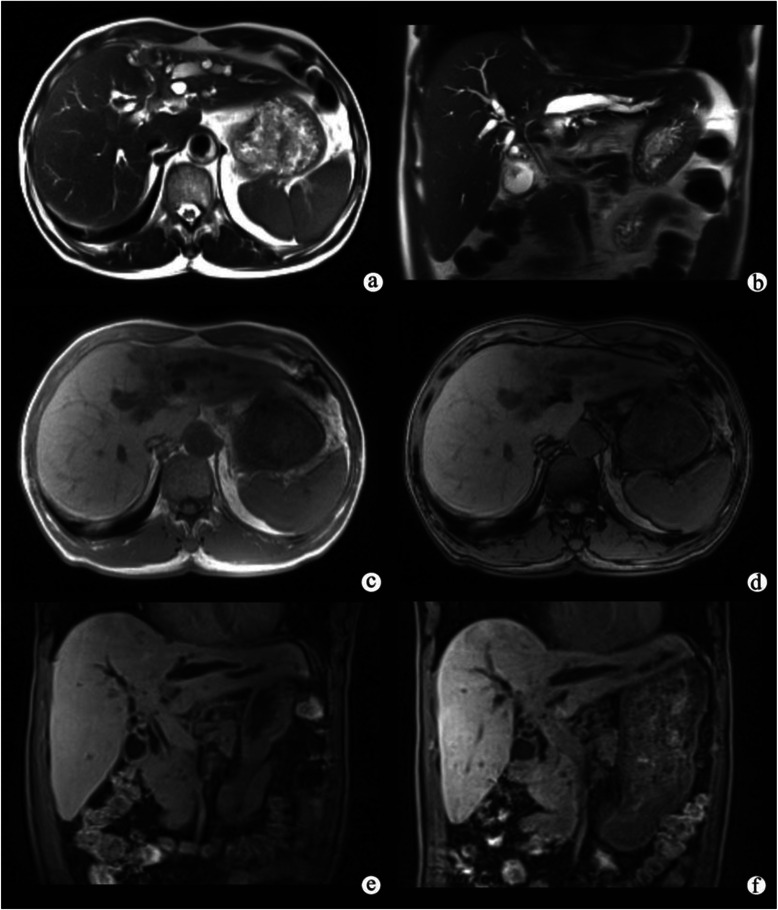
**Infiltrative hilar cholangiocarcinoma in a patient with progressive jaundice.** A axial **(a)** and coronal **(b)** T2-weighted image showed wall thickening of bile duct at the hepatic hilar level, and the left hepatic ducts were dilated. In **(c)** and out **(d)** of phase imagings showed no change on the signal intense of liver background. The hepatocellular phase **(e)** showed nearly no Gd-EOB-DTPA in the common bile duct. The biliary phase **(f)** showed that there was little Gd-EOB-DTPA in the common bile duct, indicating that the function of biliary system was abnormal.

**Figure 3:**
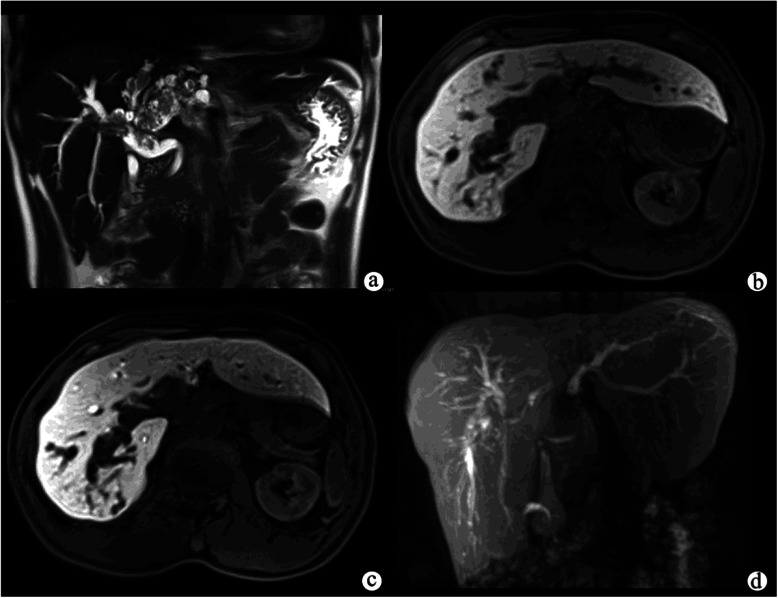
**Papillary cystadenocarcinomain a patient with right abdominal pain.** A T2-weighted image **(a)** showed marked dilatation of the intrahepatic and extrahepatic bile ducts with multiple heterogeneously hypointense polypoid nodules on the surface. The hepatocellular phase **(b)** showed that the polypoid nodules were homogeneously hypointense, and nearly no Gd-EOB-DTPA was seen in the dilated bile duct. The biliary phase **(c)** showed that most of the dilated bile duct was filled with Gd-EOB-DTPA, indicating that the function of biliary system was normal. Gd-EOB-DTPA-enhanced MR cholangiography **(d)** showed that the intrahepatic and extrahepatic bile ducts were well-filled with contrast agent.

Exophytic (mass-forming) tumors (Figure 1) were found in 10 (40%) patients, infiltrative (periductal) tumors (Figure 2) in 10 (40%) patients, polypoid (intraductal) tumors (Figure 3) in two (4%) patients, and combined tumors (two admixed growth patterns) in three patients. All the exophytic tumors and polypoid tumors were well-defined and rounded; while the infiltrative tumors had ill-defined margins, and combined tumors were a mixture of both. Their size ranged from 0.5 to 16.3 cm in transverse diameter (mean 5.2 ± 0.4 cm). The tumors involved the right hepatic lobe (52%, n = 13), the left hepatic lobe (20%, n = 5), and both lobes (28%, n = 7). On T2WI, the tumors were hyperintense relative to normal livers (Figure 1a, and Figure 2a, b) in 15 of 25 (60%) patients, hypointense in three patients (12%) (Figure 3a), and had mixed intensity in seven patients (28%). All the subjects did not demonstrate evidence of diffuse liver diseases such as hepatic steatosis and cirrhotic morphology on in and out of phase imaging (Figure 2c,d),

On dynamic MR imaging, 23 of 25 (92%) patients had minimal or moderate contrast enhancement at the periphery of the tumor on AP, with progressive or concentric filling on PVP and EP. The other two (8%) patients had papillary cystadenocarcinomas that were homogeneously enhanced during all dynamic phases. On hepatobiliary MR imaging, 22 of 25 (88%) patients demonstrated heterogeneously hypointense appearances (Figure 1b, c and Figure 2e, f); and three (12%) patients demonstrated homogeneously hypointense apperances (Figure 3b, and c) compared with the surrounding liver parenchyma, which showed strong enhancement. The liver parenchyma of three (12%) patients showed heterogenous enhancement on HP (Figure 1c) and BP, with the liver parenchyma around the tumor enhancing less than that farther away from the tumor. Bile duct dilatation was seen in 23 (92%) patients, and satellite nodules were present in six patients (24%). Invasion of the portal vein branches and focal liver atrophy were seen in four (16%) patients.

In addition, patients in the NTB group on BP (10 exophytic tumors and two polypoid tumors) had clear visualization of the whole biliary system, including the common bile duct, common hepatic duct, left and right hepatic ducts, sub-branches of the intrahepatic bile ducts, cystic duct, and the gallbladder (Figure 1c and Figure 3b, c, d), while patients in the ETB group on BP (10 infiltrative tumors and three combined tumors) had indistinct or even no visualization of the biliary tree (Figure 2b, c).

### Quantitative image analysis

**Figure 4:**
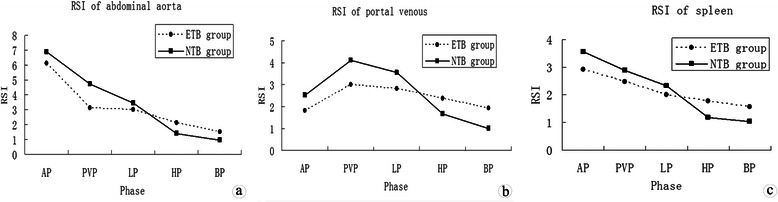
**Patients with normal TB levels (NTB group, solid line) had higher relative signal intensities (RSIs) of the abdominal aorta (a), portal veins (b) and spleen (c) than those with elevated TB levels (ETB group, dotted line) on AP, PVP, and EP, and lower RSIs on HP and BP.**

**Figure 5:**
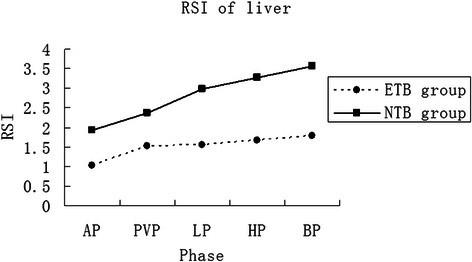
**Patients with normal TB levels (NTB group, solid line) had higher RSIs of the liver parenchyma than those with elevated TB levels (ETB group, dotted line) on all phases.**

There were 12 patients in NTB group (mean TB level 11.0 ± 1.3 μmol/L) and 13 patients in ETB group (mean TB level 85.2 ± 3.7 μmol/L). Figure 4a, b and c, Figure 5, and Table 1 show the time-courses of the changing mean relative signal intensities of the abdominal aorta, portal vein, spleen, and liver in these groups. The RSI_A_, RSI_V_ and RSI_S_ of the NTB group on AP (*t* = 7.731, 7.528 and 4.003, respectively; *P* < .001), PVP (*t* = 17.603, 9.761 and 3.965, respectively; *P* < .001), and EP (*t* = 4.554, 8.005 and 2.976, respectively; *P* < .001) were significantly higher than those of the ETB group. However, the RSI_A_, RSI_V_ and RSI_S_ of the NTB group on HP (*t* = −7.158, −8.020 and −5.480, respectively; *P* < .001) and BP (*t* = −9.023, −8.319 and −4.207, respectively; *P* < .001) were significantly lower than those of the ETB group (Table 1). The RSI_L_ of the NTB group on all phases (*t* = 8.441, 6.403, 11.518, 13.362 and 14.962, respectively; *P* < .001) were significantly higher than that of the ETB group.

**Table 1:** **Mean relative signal intensities of both groups (Mean Values ± SD)**

	AP	PVP	EP	HP	BP
RSI_A_					
NTB group	6.87 ± 0.24	4.72 ± 0.21	3.43 ± 0.23	1.40 ± 0.18	0.95 ± 0.16
ETB group	6.12 ± 0.24	3.11 ± 0.24	2.98 ± 0.27	2.09 ± 0.28	1.51 ± 0.15
*t*	7.731	17.603	4.554	−7.158	−9.023
*P* value	<.001	<.001	<.001	<.001	<.001
RSI_V_					
NTB group	2.53 ± 0.27	4.12 ± 0.29	3.57 ± 0.25	1.67 ± 0.20	1.01 ± 0.27
ETB group	1.83 ± 0.20	3.00 ± 0.28	2.82 ± 0.21	2.38 ± 0.24	1.93 ± 0.28
*t*	7.528	9.761	8.005	−8.020	−8.319
*P* value	<.001	<.001	<.001	<.001	<.001
RSI_S_					
NTB group	3.56 ± 0.48	2.89 ± 0.28	2.33 ± 0.23	1.17 ± 0.27	1.03 ± 0.29
ETB group	2.90 ± 0.35	2.46 ± 0.26	2.01 ± 0.30	1.77 ± 0.27	1.57 ± 0.34
*t*	4.003	3.965	2.976	−5.480	−4.207
*P* value	<.001	<.001	<.001	<.001	<.001
RSI_L_					
NTB group	1.94 ± 0.26	2.36 ± 0.38	2.99 ± 0.30	3.27 ± 0.31	3.56 ± 0.28
ETB group	1.01 ± 0.29	1.53 ± 0.27	1.56 ± 0.32	1.66 ± 0.29	1.78 ± 0.31
*t*	8.441	6.403	11.518	13.362	14.962
*P* value	<.001	<.001	<.001	<.001	<.001

AP = arterial phase; BP = biliary phase; EP = equilibrium phase; HP = hepatocellular phase; ETB = elevated total bilirubin; NTB = normal total bilirubin; PVP = portal venous phase; RSI = relative signal intensity; RSI_A_ = RSI of the abdominal aorta; RSI_L_ = RSI of the liver; RSI_S_ = RSI of the spleen; RSI_V_ = RSI of the portal vein.

## Discussion

Several previous reports have described conventional MR findings in patients with cholangiocarcinoma [2,6-8]. Its characteristics include hypointensity on T1-weighted images, hyperintensity on T2-weighted images, the presence or absence of a central scar, ductal dilatation, satellite nodules, portal vein invasion, and lobar atrophy. In this study, intrahepatic tumors were generally mass-like, whereas extrahepatic tumors were often periductal, which concurred with previous reports [6-8].

Patients with exophytic cholangiocarcinoma rarely present with central biliary system obstruction because the tumors arise from and infiltrate along distal intrahepatic bile ducts. Consequently, these tumors are usually large at the time of clinical diagnosis. On the contrary, infiltrative cholangiocarcinomas almost always obstruct the biliary system as most tumors occur at the common hepatic duct and its bifurcation. As a result, infiltrative tumors usually present with jaundice, or patients become jaundiced shortly after the onset of right upper quadrant pain. Polypoid cholangiocarcinomas are infrequently found in both the intra- and extrahepatic ducts. The histological type of this tumor is mostly papillary adenocarcinoma characterized by its intraluminal growth [19,20]. Clinically, patients with polypoid cholangiocarcinoma can present with recurrent episodes of abdominal colic or jaundice.

The basic principle of early dynamic phase imaging with Gd-EOB-DPTA is the same as that for use of gadolinium-based, nonspecific extracellular contrast agents. On dynamic MR imaging, rim-like or band-like contrast enhancement around the tumor could be observed during the early phase, with progressive and concentric filling of contrast material during the later phase. In our study, 92% (23/25) of tumors showed progressive enhancement, similar to that described in previous reports [6,21]. The centripetal progression of enhancement was attributed to the abundant fibrous tissue in the center of the tumor. However, the two patients with papillary cystadenocarcinoma showed homogeneous enhancement during all the dynamic phases. It is hypothesized that the different enhancement patterns of cholangiocarcinoma may depend on the amount of fibrous components in the tumor, and that exophytic or infiltrative cholangiocarcinomas may carry more fibrous components than polypoid cholangiocarcinoma.

Normal liver parenchyma more avidly enhances during both HP and BP phases of Gd-EOB-DTPA-enhanced MR imaging than Gd-DTPA-enhanced MR imaging [18], and because there are no cells of hepatocytic origin in cholangiocarcinoma, all the cholangiocarcinomas in this series appeared hypointense on HP and BP images. On the contrary, cholangiocarcinomas often show delayed central enhancement on Gd-DTPA-enhanced MR images. The sharp contrast between the liver parenchyma and cholangiocarcinomas may allow more accurate assessment regarding the extent of the tumor as well as the number of lesions, and may assist in determining type of treatment and subsequent prognosis [13].

In addition, the liver parenchyma around the tumor of some patients (12%, 3/25) enhanced less intensely than that farther away from the tumor on HP and BP. This might be explained by the cholestasis caused by tumor compression. The cholestasis was brought about the alterations in hepatocyte function and thereby affected hepatocyte uptake and biliary excretion of Gd-EOB-DTPA, which weakened contrast enhancement of the liver parenchyma near the tumor. This suggests that the hepatobiliary phase of Gd-EOB-DTPA-enhanced MR imaging may allow assessment of the liver function and aid treatment planning.

In this study, we quantitatively evaluated the effect of serum TB levels on Gd-EOB-DTPA-enhanced MR cholangiography and on the relative signal intensities of the abdominal aorta, portal vein, spleen, and liver. When Gd-EOB-DTPA is injected intravenously, the contrast agent enters hepatocytes by an organic anion transport system after the vascular phase, and is excreted into the biliary system through the glutathione-S-transferase transport system [22]. Because Gd-EOB-DTPA uptake is mediated by the same transporter responsible for bilirubin transport, cirrhotic patients with a high Model for End-stage Liver Disease (MELD) score and/or an elevated serum TB level are less likely to achieve adequate Gd-EOB-DTPA-enhanced MR cholangiography for displaying biliary tree morphology [23]. As mentioned above, infiltrative cholangiocarcinomas almost always lead to biliary system obstruction, and in our study, 10 infiltrative tumors and three combined tumors with TB levels >22 μmol/L showed reduced or no visualization of the biliary tree on BP. Moreover, high level of TB resulted in reduced RSIs of the abdominal aorta, portal vein, and spleen on dynamic phases, but increased RSIs of these organs on HP and BP. In the case of the liver RSI, a high level of TB attributed to the low enhancement of the parenchyma during all the phases. This suggests that the TB level of patients with cholangiocarcinoma may play an important role in both the extracellular and intracellular kinetics of Gd-EOB-DTPA and may, to some extent, be associated with the morphologic type of cholangiocarcinoma.

On the other hand, impaired biliary function would be suspected in cholangiocarcinoma patients with altered RSIs of the abdominal organs or reduced or absent visualization of the biliary tree after administration of Gd-EOB-DTPA. To our knowledge, this is the first study that has quantitatively evaluated the effect of TB levels on the biliary excretion of Gd-EOB-DTPA in patients with cholangiocarcinoma.

We acknowledge the following limitations in this study: Firstly, the number of patients was relatively small, especially the number with polypoid type and combined type tumors. Secondly, the retrospective nature of the study might have given rise to selection bias.

## Conclusions

Our study has demonstrated the spectrum of appearances of different types of cholangiocarcinomas on Gd-EOB-DTPA-enhanced MR imaging. In addition, it showed that cholangiocarcinoma patients with elevated TB levels have delayed excretion of the hepatobiliary contrast agent Gd-EOB-DTPA compared to patients with normal TB levels, and that the RSIs of the abdominal aorta, portal vein, spleen, and liver can reflect underlying biliary function.

### Ethics approval

The ethics approval was provided by The First Affiliated Hospital, Sun Yat-Sen University, China.

## Abbreviations

RSI: Relative signal intensities
TB: Total bilirubin
A: RSIs of the abdominal aorta
V: Portal vein
L: Liver
S: Spleen
HP: Hepatocellular phase
BP: Biliary phase
